# Clocks, Rhythms, Sex, and Hearts: How Disrupted Circadian Rhythms, Time-of-Day, and Sex Influence Cardiovascular Health

**DOI:** 10.3390/biom11060883

**Published:** 2021-06-14

**Authors:** O. Hecmarie Meléndez-Fernández, James C. Walton, A. Courtney DeVries, Randy J. Nelson

**Affiliations:** 1 Department of Neuroscience, Rockefeller Neuroscience Institute, West Virginia University, Morgantown, WV 26505, USA; james.walton@hsc.wvu.edu (J.C.W.); randy.nelson@hsc.wvu.edu (R.J.N.); 2 Department of Medicine, Division of Oncology/Hematology, West Virginia University, Morgantown, WV 26505, USA; courtney.devries@hsc.wvu.edu; 3 West Virginia University Cancer Institute, West Virginia University, Morgantown, WV 26505, USA

**Keywords:** circadian rhythms, circadian disruption, cardiovascular system, sex differences, time-of-day, chronomedicine, personalized medicine

## Abstract

Cardiovascular diseases are the top cause of mortality in the United States, and ischemic heart disease accounts for 16% of all deaths around the world. Modifiable risk factors such as diet and exercise have often been primary targets in addressing these conditions. However, mounting evidence suggests that environmental factors that disrupt physiological rhythms might contribute to the development of these diseases, as well as contribute to increasing other risk factors that are typically associated with cardiovascular disease. Exposure to light at night, transmeridian travel, and social jetlag disrupt endogenous circadian rhythms, which, in turn, alter carefully orchestrated bodily functioning, and elevate the risk of disease and injury. Research into how disrupted circadian rhythms affect physiology and behavior has begun to reveal the intricacies of how seemingly innocuous environmental and social factors have dramatic consequences on mammalian physiology and behavior. Despite the new focus on the importance of circadian rhythms, and how disrupted circadian rhythms contribute to cardiovascular diseases, many questions in this field remain unanswered. Further, neither time-of-day nor sex as a biological variable have been consistently and thoroughly taken into account in previous studies of circadian rhythm disruption and cardiovascular disease. In this review, we will first discuss biological rhythms and the master temporal regulator that controls these rhythms, focusing on the cardiovascular system, its rhythms, and the pathology associated with its disruption, while emphasizing the importance of the time-of-day as a variable that directly affects outcomes in controlled studies, and how temporal data will inform clinical practice and influence personalized medicine. Finally, we will discuss evidence supporting the existence of sex differences in cardiovascular function and outcomes following an injury, and highlight the need for consistent inclusion of both sexes in studies that aim to understand cardiovascular function and improve cardiovascular health.

## 1. Introduction

According to the most recently available Centers for Disease Control (CDC) data, cardiovascular diseases (CVDs) were the leading cause of mortality in the United States in 2019 [1], and the World Health Organization (WHO) attributed 16% of world-wide deaths to ischemic heart disease [2]. Cardiovascular diseases share multiple risk factors, of which many are modifiable, such as poor diet, lack of physical activity, and substance abuse. Another modifiable risk factor that has recently been identified is exposure to artificial light at night (aLAN). aLAN disrupts the integrity of internal circadian rhythms and deranges temporal organization of physiology and behavior (below). Rodent studies demonstrate that exposure to aLAN promotes and/or exacerbates these conditions, as well as other metabolic disturbances. Furthermore, mounting evidence from longitudinal studies of nurses’ and other night shift workers’ health has demonstrated the increased risks of developing CVDs (reviewed in [3,4]), gastrointestinal disorders (reviewed in [5,6]), metabolic disturbances [7,8], and cancer (reviewed in [9,10]). Indeed, the International Agency for Research on Cancer now considers night shift work a probable carcinogen for humans [11], highlighting the seriousness of its potential consequences. Again, exposure to aLAN disrupts individuals’ endogenous physiological rhythms that have evolved in a highly conserved and tightly regulated manner, and this disruption leads to organismal dysfunction. Thus, understanding whether exposure to aLAN links shiftwork and these disorders is epidemiologically and clinically relevant. Here, we will briefly review evidence supporting circadian rhythm influences on the cardiovascular system, and then focus on cardiac circadian rhythm (CR) disruption. We will then discuss the current gaps in cardiovascular circadian biology, specifically as they relate to the lack of reporting the time-of-day (ToD) when data were obtained, as well as failure of inclusion of both sexes.

## 2. Biological Rhythms, the Master Biological Clock, and the Mechanisms Setting and Maintaining Circadian Rhythms

Nearly all organisms on Earth have an internal, organizational, timing system termed biological oscillators or ‘clocks’. These endogenous oscillators establish and maintain rhythms that allow organisms to anticipate and prepare for predictable events such as resource scarcity or darkness, with the goal of efficiently using energy, properly allocating resources, and optimally timing behaviors, including reproduction. These clocks drive circadian (having a period of ~24 h) rhythmicity in physiology and behavior and govern most biological processes. Remarkably, these rhythms persist in cultured cells, suggesting an intrinsic component setting the pace or ‘keeping time’ [12]. Although the amplitude of cellular rhythms diminishes over time in culture, serum shock to the culture reestablishes rhythmicity [13], indicating that one or more factors contained in the serum act as time giving (*zeitgeber*) or synchronizing cues for these cells. These are two fundamental characteristics of the rhythms, (1) persistence in the absence of external cues, and (2) re-entrainment with exposure to an external *zeitgeber* [14,15,16]. Lesion studies of the paired suprachiasmatic nuclei (SCN) of the hypothalamus established this region as the ‘master clock’ regulator of the body [17,18,19]. Extensive subsequent studies have further characterized the molecular clockworks, as well as additional circadian processes and pathways that are regulated by the SCN [20].

Organisms evolved with distinct activity patterns, irrespective of their temporal niche, that are aligned with relatively brightly illuminated days and dark nights. Although other entraining cues exist, light is the most potent *zeitgeber* for circadian rhythms. In mammals, the SCN receives photic information from the retina [21] via the monosynaptic neural pathway of the retinohypothalamic tract [22,23], which projects from activated intrinsically photosensitive retinal ganglion cells (ipRGCs) [24] expressing melanopsin [25,26]. Melanopsin is a photopigment that is preferentially activated by short wavelength light, and is responsible for converting photic energy into a neural signal, which relays non-image-forming visual information to the brain [27,28]. The ipRGCs are especially sensitive to short (blue) wavelength light (~460–480 nm). Exposure to aLAN in this range inhibits melatonin release and resets the molecular clock [29,30,31].

Additional regions innervated by the RHT, in addition to the SCN, include the olivary pretectal nuclei, the dorsal lateral geniculate nuclei, and the superior colliculi, which command pupillary control, govern responses to ambient light, and provide some spatial information [27,28,32]. Upon photic activation of the SCN, a signaling cascade induces the transcription of the core clock proteins’ circadian locomotor outputs kaput (CLOCK), cryptochrome (CRY), period (PER), and brain and muscle aryl hydrocarbon receptor nuclear translocator-like protein 1 (BMAL1) which are expressed in an oscillatory pattern over a 24 h period, in a transcriptional translational feedback loop (TTFL) [33,34]. Briefly, transcriptional activity begins at the start of the circadian cycle with heterodimerization and translocation to the nucleus of the transcription factors BMAL1 and CLOCK. This heterodimer binds to E-box elements of the target genes *Cry* and *Per*. Following an accumulation of these gene products, they dimerize in the cytoplasm and eventually translocate into the nucleus. Upon entry, they inhibit E-box transactivation by BMAL1 and CLOCK, ultimately inhibiting their own transcription. Degradation and depletion of PER and CRY proteins remove the ‘brakes’ on *Per* and *Cry* transcription, and the cycle begins anew, ending (and beginning) the cycle of ~24 h [35]. Post-translational modifications, such as phosphorylation, alter core circadian protein activity, stability, and subcellular localization, to ensure their function and availability in the necessary location at the appropriate time of the cycle. A secondary interlocked loop is also activated by the BMAL1:CLOCK heterodimerization. REV-ERBα,β repressors disrupt the stimulation of BMAL1 and CLOCK transcription through RORα, RORβ, and RORγ activators. These circadian TTFLs regulate the activity of ~43% of all transcribed genes [36], which are themselves expressed in a circadian fashion in the various organs and cells.

## 3. Entrainment and Disruption of the SCN Clock

As mentioned, light is one of the primary *zeitgebers* for the master clock, but others, such as food [37] and exercise [38,39,40], have also been well characterized. The relationship among these *zeitgebers* is not unidirectional, as the internal clock directs biological processes through the production and secretion of hormones, neurotransmitters, and other signaling molecules, but the presence of these external cues can also reset the internal clock. For instance, food consumption throughout the day is regulated by coupling peripheral clocks to the master clock, which modulates the cyclical balance of hormones, such as leptin and ghrelin [41,42,43] (an internal cue). However, if food intake (an external cue) is time-restricted, then the clocks will become uncoupled, and the feeding rhythm reset [44,45,46,47,48].

The now ubiquitous presence of aLAN has allowed us to extend our daily activities into the night. However, humans and all other vertebrates evolved with an internal timing system aligned with the 24 h illumination cycles of bright days and dark nights. Consequently, a challenge to the physiological and behavioral organization of their internal clock can negatively influence their well-being and survival [49,50,51,52,53]. Indeed, disruption of the molecular clock and its rhythms impairs cognition [54] and metabolism [53,55,56,57,58], alters normal vascular function, and increases the risk of CVDs [3,4], gastrointestinal disorders [5,6], metabolic disturbances [7,8], and certain cancers [9,10]. The occurrence and increased incidence of these maladaptive processes coincides with technological and societal advances that expose individuals to light ‘around the clock’. This is the case with night shift workers, whose activity pattern is typically misaligned with their internal clock, and who are often exposed to bright light during the night, further disrupting their biological rhythms.

## 4. SCN Control of Peripheral Clocks

In addition to the central clock in the SCN, individual organs and cells outside the SCN rhythmically express core clock genes. These are termed peripheral clocks. Individual cells contain autonomous clocks; nonetheless, cells work in concert to time the occurrence of physiological events optimally, from gene transcription to physiological and behavioral outputs, and every process in between. The SCN regulates peripheral clocks both through indirect and direct mechanisms. Indirectly, it achieves this by mediating the release of neural and humoral signaling factors, as well as by modulating the expression of circadian clock genes. Exogenous events, such as feeding [47] and exercise [59], can also provide cues to reset peripheral clock rhythms in the liver [60,61], or in cardiomyocytes, for example, by reversing the light cycle and, consequently, the circadian phase dictated by light, or in vitro, through serum shock exposure [62]. Another mechanism of SCN-mediated peripheral clock regulation is by direct modulation of the autonomic nervous system, as described in the next section.

## 5. Circadian Rhythms, Autonomic Function, and the Cardiovascular System

Circadian control of vascular and endothelial function, both centrally and peripherally, is well established [4]. Proper daily alignment of vascular function relies on a precisely regulated balance between parasympathetic and sympathetic branches of the autonomic nervous system to modulate heart rate [63] (HR), heart rate variability [64] (HRV), vascular tone, blood pressure (BP), and endothelial function. This organization allows the vascular system to produce the necessary factors and mediators, such as prothrombotic and antithrombotic factors, and nitric oxide (NO), at the appropriate time of the day; this temporal organization supports optimal physiological function during the increased demands of the organisms’ active period, while optimizing recovery and replenishment during the inactive phase. Dysregulation of this balance appears to lead to cardiovascular pathologies (discussed below) that can be exacerbated by both endogenous and exogenous risk factors. Prior to the onset of locomotor activity, BP [65] begins to increase; in humans, elevated BP typically occurs between ~06:30 and 08:00 h, coinciding with the increased expression of clotting factors [66], platelet production, and thrombogenesis [67,68,69]. BP begins a gradual decrease in the afternoon, reaching the trough during the night; failure to decrease BP during the night is likely a result of the lack of vagal tone regulation by sympathetic and parasympathetic systems. Moreover, the increased expression of Plasminogen Activator Inhibitor-1 (PAI1) during the morning (peaking ~06:30 h) [67], in conjunction with enhanced platelet function between 08:00–12:00 h [68], increased levels of circulating fibrinogen and other clotting factors, as well as the decreased fibrinolytic activity during these early hours [69], increases the risk of thrombosis. Non-dipping BP status, in conjunction with the rhythm of expression of these factors, places patients at an increased risk of pathological cardiac events during the morning [70].

Daily rhythms in molecular regulators of vascular function have also been reported in mice. The endothelial membrane, which lines the heart and blood vessels, primarily controls factors that mediate vascular relaxation, such as nitric oxide (NO), or contraction, such as thromboxane and endothelin-1 [71]. Thrombomodulin (TM), a glycoprotein primarily found in vascular endothelial cells (VECs), and which plays a role in the regulation of intravascular coagulation, is upregulated by the circadian genes *Clock* and *Bmal2* [72]. TM exhibits rhythmic expression in the heart and lung, with a peak at ZT18, and trough at ZT6; the deletion of *Clock* abolishes this rhythm in mice [72].

Fatty acids function as fuel for cellular function, and a balance between fatty acid availability and oxidation is critical in maintaining proper metabolic function within skeletal muscles, the pancreas, and the liver to avoid the development of metabolic risk factors. *Clock* homozygous mutant mice are characterized by a phenotype resembling metabolic syndrome, a cluster of cardiovascular risk factors including obesity, dyslipidemia, hypertension, insulin resistance, and hyperglycemia [73]. Further, cardiomyocyte-specific *Clock* mutant mice significantly altered the expression of genes encoding myocardial fatty acid response proteins [62]. Taken together, these data support the hypothesis that cardiomyocyte circadian genes are necessary for proper metabolic functioning. Other studies have documented similar fluctuations in clock gene expression and/or regulation in VECs and vascular smooth muscle cells (VSMCs) [72,74,75].

Rhythms in vascular function are also observed at the molecular level. RNA sequencing data indicate that 6% and 4% of protein-coding genes in the heart and aorta, respectively, are transcribed in a circadian fashion [36], encompassing genes that control the regulation of the expression of cardiovascular-specific cells, their signaling, metabolism, and cardiac contractility [76]. Indeed, gene expression analysis in the heart and liver showed that more than 8–10% of the genes expressed in these organs exhibit circadian expression, although many of these are tissue specific, with only 37, of more than 12,000 genes assayed, overlapping between these organs [77]. Rodent studies have examined cardiac clock gene function by manipulating these genes in the heart, aorta, VECs, and VSMCs (Table 1). Mutation of the clock gene *Per2* alters aortic endothelial function by reducing NO production, decreasing vasodilatory prostaglandins, and increasing vasoconstrictors, which result in impaired endothelium-dependent vasodilator responses [78]. Global knockout (KO) of peroxisome proliferator activated receptor-γ (PPAR-γ), a putative activator of BMAL1 in vasculature, decreases the variation in diel heart rate [79] and abolishes the ability to regulate mean arterial heart rate and pressure, while maintaining clear locomotor activity differences between day and night [80]. A germline deletion of *Bmal1* abolished 24 h fluctuations in heart rate and BP, without affecting sleep/wake or fast/feed cycles [81], disrupted metabolism, and impaired contractile function [82]. Notably, these *Bmal1* KO mice also display extensive cardiac damage from an early age, reminiscent of aged animals [83]. Further effects on vascular function regulation are observed in mice lacking other components of the core clock. For instance, *Cry1/2* double deletion gives rise to salt-sensitive hypertension [84], whereas *Per1* deletion protects female, but not male, C57BL/6J mice from a non-dipping hypertension phenotype [85]. Further, *Per1* was implicated in aldosterone [86] and sodium regulation [87], both of which play a role in BP maintenance. Taken together, these data suggest that dysregulation of core clock components can negatively affect cardiovascular health by impairing proper endothelial function, dampening the physiological amplitude of BP [88], disrupting sodium homeostasis, and promoting a senescent cardiac phenotype. 

In vitro studies also confirm rhythmicity in the cardiac system. Cultured rat cardiomyocytes show rhythmic gene expression of clock genes *Bmal1, Rev-Erba*, and *Per2*, and both in vivo and in vitro studies show rhythmic oscillations in clock gene transcripts for *Per2*, and *Bmal1* in mouse aorta and vascular tissue [74,89]. Cultured explants of heart, veins, and arteries demonstrated rhythmicity, albeit not in phase with that found in vivo at the time of explantation (the removal of live tissue for culture), but in phase with the time of culture [89]. These data demonstrate that the cardiac tissue in culture maintains an internal clock, but not phase relationships with other tissues.

Other than through genomic analyses, there are few techniques that can be used to study human genetic or molecular cardiovascular circadian rhythms in vivo, and, as such, a very limited number of studies have been published on this topic. Nonetheless, Leibetseder and colleagues [90] obtained myocardial samples during orthotopic heart transplants of 64 patients. They observed rhythmic clock gene mRNA expression in the human heart, with *BMAL1* peaking in the evening, and *PER1* and *PER2* peaking in the morning, which coincides with the time of day of most frequent myocardial infarctions. They did not observe a circadian rhythm of expression in *CRY1*. Finally, they noted that human cardiac gene expression phases were antiphase to those of rodents, which was expected, as these coincide with their respective activity phases. These data are complemented by a meta-analysis by Hughey and Butte [91], in which they determined that clocks outside the SCN (i.e., peripheral tissue) are phase shifted by ~12 h between diurnal and nocturnal species. However, they reported that the master clock (SCN) phasing of both diurnal (human) and nocturnal (mice) species did not show the same antiphasic rhythm. Taken together, these data suggest that the temporal components of the master clock are conserved across species, and it is the peripheral clocks that differ in the phase of molecular dynamics based on the temporal dynamics of the species. Consequently, it is reasonable to conclude that although the SCN is entrained by and to the external environment, yet other (peripheral) clocks are more aligned with the organism’s active phase, likely to optimize efficiency to meet elevated metabolic demands during activity and recovery during rest. Additionally, humoral and neural signaling that inherently take place during the active (or inactive) phase may also influence peripheral clocks and uncouple them from the central clock; however, the precise mechanisms by which the peripheral phases of diurnal and nocturnal species diverge from the phase of the SCN are still largely undescribed [92].

Another interesting outcome of the study by Leibetseder et al. [90] was the observation of the elevated difference in amplitude of cardiac expression of clock genes. Prior studies in other tissues have documented marked differences (~30–100%) in peak and trough expression of these genes; however, in human cardiomyocytes, the difference between the peak and trough for PER1 was ~200%, and for BMAL1 was ~450%. The authors propose that these differences may be attributed to elevated metabolism in cardiomyocytes or low rates of differentiation; nonetheless, the underlying explanation for this difference remains unspecified.

The results obtained from these studies provide insight into the molecular signaling and interactions among the (human) central and peripheral clocks. However, additional questions arise. For instance, is it probable that behavioral or anthropogenic factors, such as caffeine and/or alcohol consumption, exposure to artificial light at night, or other such known master clock disruptors differentially affect peripheral clocks so as to uncouple these from the SCN? Alternatively, can these behaviors or events uncouple the clocks in the associated brain regions from the temporal integration within the SCN and, in turn, uncouple the SCN from the peripheral clocks? Data suggest that this is a possibility [91]. The field of cardiac circadian biology is ever-growing, and the knowledge obtained from rodent studies has proved invaluable; however, the need for additional studies leveraging human samples is profoundly needed.

## 6. Disrupted Circadian Rhythms and Cardiac Pathology

Circadian rhythmicity strongly influences cardiovascular function. Diel variations in the onset of myocardial infarctions [93,94,95,96,97] and other cardiac events [98,99,100,101,102] have been well documented, and risk factors for cardiovascular dysfunction coincide with circadian rhythm misalignment [3,4,103]. Further, as discussed above, genetic disruption of core circadian gene expression impairs cardiovascular function and in some instances, also impairs other organ system functioning. As a result, various pathological cardiac events occur in a predictable manner, primarily during the onset of activity.

In humans, potentially fatal cardiovascular events, such as myocardial infarctions (MI) [104,105], sudden cardiac death [104], and atrial [106] and ventricular fibrillation [107,108], generally occur in the morning and have been well documented. Although these cardiovascular events can display bimodal peaks in occurrence across the day, the largest cluster generally occurs in the morning (e.g., [106]) following a circadian pattern which coincides with the elevation in platelet and prothrombotic factor production, and a decrease in cardiac endothelial function. BP also follows a distinct pattern, typically peaking during the morning hours, gradually decreasing in the afternoon, and reaching a trough during the night [109]. Although this general circadian BP pattern can vary with age, it is generally the sharp change in blood pressure that is associated with cardiovascular events. However, failure to achieve a normal decrease in nocturnal BP results in non-dipping hypertension and is a risk factor for subsequent development of CVDs. Non-dipping hypertension is likely a result of reduced vagal tone regulation by sympathetic and parasympathetic systems. Grassi and colleagues [110] reported that patients with reverse dipping had elevated early-morning sympathetic activity, presumably due to the failure to decrease sympathetic activity through the night. In sum, the simultaneous desynchrony between sympathetic-parasympathetic regulation and thrombolytic-prothrombotic fluctuations, coupled with the elevation in morning BP, renders individuals particularly vulnerable to pathologic cardiac events during these hours. Nonetheless, these effects can vary by age and be exacerbated by pre-existing conditions.

The cardiovascular system is at the core of overall organismal functioning; it provides oxygen and nutrients to the body, and aids in waste removal. When the vascular system is compromised, other cardiometabolic risk factors and comorbidities, such as metabolic syndrome, can emerge and predispose the organism to develop more serious, and potentially life-threatening, conditions. As evidence linking the occurrence of cardiovascular events to predictable times of the day mounts, and the circadian expression and circulation of specific components of the vascular system are identified, the case for circadian rhythmicity at the center of cardiovascular pathology continues to be strengthened. Further, many of the risk factors for metabolic syndrome are themselves influenced by circadian-regulated processes, such as changes in BP, glucose uptake, fatty acid oxidation, and sleep, and failure to undergo these daily rhythmic variations promotes their development and that of their associated disorders. Indeed, the circadian nature of these events has prompted the proposal of circadian disruption as an important etiological factor underlying metabolic syndrome and the suggestion that it should be renamed ‘circadian syndrome’ [111].

**Figure 1:**
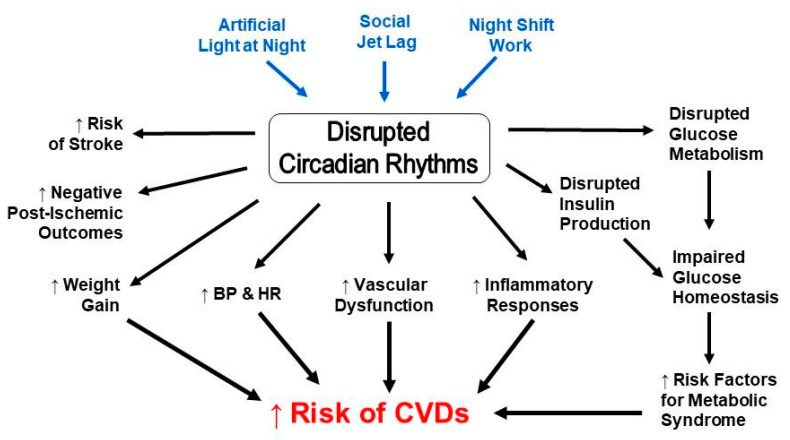
Circadian disruption and risk factors for cardiovascular diseases are interconnected.

As a result of circadian governance, multiple biological processes occur concurrently or at specific times in relation to each other, in order to maintain physiological balance. Oftentimes, these processes are interdependent of each other and share signaling factors and pathways, which, upon dysregulation in one, will result in a desynchronization in the other, in turn leading to an elevated predisposition to other malignancies (Figure 1). For instance, sleep apnea has been associated with CR disruption and increased risk of cardiovascular disease, congestive heart failure, and stroke [112,113], all of which also have a circadian component to them, and their risk factors are also associated with CR disruption. Type 2 diabetes mellitus, a condition of inadequate glucose homeostasis, is largely dependent on insulin secretion from pancreatic β-cells, which also have intrinsic clocks [114,115], and are thus highly influenced by daily changes. Moreover, CR disruption has been linked to impaired glucose metabolism [116], weight gain [117], and other metabolic processes [118], all contributors to glucose homeostasis, which, in turn, are themselves part of or contribute to metabolic syndrome, the primary cluster of risk factors for CVD. Similarly, some of these processes, and their disruption, contribute to cardiac pathology and dysfunction, and are deeply entangled within other physiological processes. One such process is immune reactivity.

Immune system activation is also regulated by circadian rhythms and may contribute to exacerbated cardiac events during the morning. During this time of day, the immune system is primed to combat infections, whereas repair and renewal functions of the system occur during the onset of the inactive phase [119]. Circadian rhythmicity in immune cells can be antiphase-dependent upon where they are measured. For example, the peak in circulating immune cells occurs during the early resting phase, whereas recruitment into tissues peaks during the active phase [120]. Indeed, cardiac neutrophil recruitment is enhanced early in the active phase resulting in worse outcomes for infarcts; these poor outcomes include increased inflammatory response, greater infarct size, decreased survival, and exaggerated post-MI left ventricular remodeling and dysfunction, when compared to infarcts occurring during the inactive phase [121]. However, expression of a CXC chemokine receptor 2 (CXCR2) antagonist or a neutrophil-specific CXCR2 knockout, which reduces the exaggerated neutrophil recruitment into the heart, inhibits these enhanced negative effects [121]. Indeed, disrupting the circadian rhythmicity of immune cells may juxtapose the infection-combating function with the repair and replenishment duties of the immune system to exacerbate the injury.

In sum, the cardiovascular system functions in a circadian fashion with daily variations in the occurrence of pathological cardiac events. Genetic studies have demonstrated that disruptions to the internal modulators of these endogenous rhythms can have dire consequences for the organism’s health and survival; nonetheless, environmental disruptors of endogenous rhythms have been understudied.

## 7. Sources of Disrupted Circadian Rhythms

Circadian rhythms can be entrained by various external cues, and, as such, these cues can be the targets of rhythm disruptors. Although various potential sources of circadian disruption exist, in this review we will only focus on environmental lighting, night shift work, and social jetlag. We focus on these three sources because they are ubiquitous, are socially and clinically relevant, have been widely reported, and can be modified with relative ease.

### 7.1. Environmental Lighting

As mentioned, organisms evolved on Earth with an internal timing system aligned with bright days and dark nights. The invention of electric lighting nearly 150 years ago initiated a revolution, but also the end of the once-completely dark nights. Artificial light now inundates the world with a night sky glow known as ‘light pollution’. Light pollution is defined as the alteration of natural night light caused by anthropogenic sources of light [122,123]. According to Falchi and colleagues [123], 80% of the world and over 99% of Europe and the United States live under polluted night skies. Sources of artificial light include vehicles, buildings, street and traffic lights, among others. Upon their invention, incandescent light bulbs emitted light of a full spectral composition. Technological advances have allowed us to develop more cost- and energy-efficient sources of illumination, the light-emitting diodes (LEDs), but their spectral composition has negative consequences on the environment [124] and mammalian circadian rhythms. LEDs emit light with a peak in the short-wavelength of the spectrum, which, as described above, is the wavelength range that activates the photoreceptive pathway that relays information to the master clock in the SCN. Activation of this pathway at inappropriate times of the day (night time) may reset the clock and lead to circadian misalignment.

### 7.2. Night Shift Work and Social Jetlag

As a result of industrialization and technological advances, the ‘day’ now extends well past the daylight hours. Much of the workforce has deviated from the traditional, so-called ‘9 to 5′ workday and has incorporated non-standard work shifts such as nights and split shifts. American and European surveys reported that between 15 and 30% of the population engage in some kind of night shift work [125]. Although beneficial in many aspects, such as increased job availability and productivity, around-the-clock care in hospital settings, and others, multiple longitudinal studies have demonstrated that night shift work results in circadian misalignment, causing these new ‘workdays’ to have potentially noxious consequences on health [7,9,10] and, more specifically, on cardiovascular and metabolic diseases [7,8,126,127,128]. Indeed, one example is altered BP rhythms. Kitamura and colleagues [129] reported a change in BP dipping status in night shift workers. After one night of night shift work, day-night differences in BP were reduced by 8.5%, but after four days, the BP status reversed. However, these participants (night shift workers), worked a four-day night shift, two days off, four-day day shift schedule. Thus, continued alternation between day and night shifts has the potential to perpetuate the disrupted rhythm in BP by reducing the amplitude of the physiological rhythm, which can lead to more serious health issues, such as stroke [130]. These data, together with altered sleep [125,131,132,133] and eating [134,135,136] patterns, along with the health and performance issues that may arise because of these [131,137,138], highlight the deleterious effects of circadian disruption by night shift work.

Similarly, social jetlag (SJL) disrupts daily rhythms and results in circadian misalignment. SJL is defined as the difference in sleep timing between work and free days as a consequence of the discrepancy between the individual’s circadian rhythm and the social clock [139,140]. Thus, it is common for people to shift their wake-sleep and other circadian rhythms 3–6 h in both directions every weekend voluntarily. The increasing demands of school and work and the abundance of nightlife options extend our waking hours and, in turn, alter both our sleep time and length. Both chronic shift work and SJL uncouple central and peripheral clocks, and impair functioning. In the context of cardiovascular health, these increase the risk of developing CVDs [7,141,142] and unhealthy behaviors such as smoking and consuming high amounts of caffeine [143], further contributing to cardiovascular risk.

Experimental models of shiftwork have been developed to address how this mode of circadian disruption affects humans. One of the prevalent models of circadian disruption emulates shift work by altering behavioral and/or environmental cues to misalign the daily rest-activity patterns with the biological day. Morris and colleagues [70] implemented one such circadian misalignment protocol similar to what shift workers experience to assess biomarkers of common risk factors of cardiovascular disease [144,145,146,147]. In these otherwise healthy individuals, they observed increased BP, primarily during sleep periods, increased inflammatory markers, and decreased awake cardiac vagal tone by 8–15%. Increased daily BP and decreased vagal tone are clinically relevant and may be indicative of a pathological state, as high BP is a risk factor for CVDs [70,148,149,150], and decreased vagal tone is typically observed with aging. Furthermore, decreased vagal modulation may not adequately counterbalance sympathetic stimulation, which can ultimately lead to ventricular tachycardia or sudden cardiac arrest [151,152] and, as a well-established marker for heart failure [153], serves as a strong predictor of cardiac mortality following myocardial infarction [154]. Conversely, higher resting vagal tone is associated with increased stress resilience [155,156,157] and optimal cardiovascular health. Taken together, these data emphasize the precise regulation that must continually occur in the cardiovascular system to ensure homeostasis, as both too much or too little circulatory pressure can lead to pathological states.

## 8. Time-of-Day as a Biological Variable Influencing Cardiovascular Function

As discussed, observations that various pathological cardiovascular events typically occur at higher frequencies during specific times of the day [158,159,160,161,162,163], along with pioneering work focused on the daily fluctuations in BP (reviewed in [164,165,166], provide evidence for biological rhythms in the cardiovascular system. Thus far, we have briefly discussed these rhythms—how they manifest in the cardiovascular system and how disrupted rhythms can promote cardiac pathology. Considering the health implications of misaligned circadian rhythms in the cardiovascular system to both human and nonhuman animals, the need to understand how cardiovascular function and circadian rhythms influence one another to modulate overall health is important. As these data are obtained, therapeutic approaches should be developed that maximize benefits by therapy delivery when individuals best respond. This specialized intervention, referred to as chronopharmacology, chronotherapeutics, or chronomedicine, has increasingly become a pillar of personalized medicine [167,168], aiming to tailor treatment for each individual’s specific needs. However, two barriers remain in the development and establishment of this aspect of personalized medicine. First, published data rarely report circadian information as it pertains to the experimental design, and methods sections rarely provide basic temporal information from which (potentially) to extract time-of-day data. Second, interindividual circadian rhythm variation exists and must be taken into consideration when applying chronotherapeutics, as determining an individual’s endogenous rhythms requires undergoing rigorous testing conditions, which are currently impractical. In the following section, we discuss how we propose that this barrier be addressed in the literature.

Circadian rhythms are phylogenetically conserved, from fruit flies to humans [4], and exist in virtually every living organism. Hence, it does not come as a surprise that pathological events also present with some sort of daily rhythm. Indeed, human pathological cardiovascular events have confirmed this as they have a higher incidence during the morning, coinciding with alterations in other circadian-controlled physiological events, and we have discussed some of the factors we currently know that contribute to this. Animal models have allowed us to understand how the period following morning awakening contributes to these results. For instance, in a nocturnal murine model of MI, researchers demonstrated that when an ischemia/reperfusion (I/R) event occurs at the transition from the inactive to active phase (ZT12), infarct size is 3.5× larger, and exhibits greater scarring and overall tissue damage and remodeling, as well as reduced contractile function, as compared to an I/R event occurring during the active phase [169]. Further, ToD of MI onset affects healing, at least partly, through altering oscillations in cardiac neutrophil recruitment [121]. This suggests that the ability of cardiomyocytes to respond or regulate the response to injury may be dependent on ToD. Indeed, ToD effects following I/R were eradicated in mice with a non-functional cardiac clock [169], suggesting that these effects are intrinsic to the cardiac clock, and not systemically or centrally controlled.

Cardiovascular health research is testing cardioprotective measures to translate knowledge from the bench to the clinics. Although chronotherapy is a common treatment for dysregulated BP [170,171,172], circadian rhythms and ToD influencing the onset and development of cardiac pathologies are rarely considered. Indeed, ToD is infrequently reported or even taken into account in studies aiming to understand cardiovascular physiology. Aside from the aforementioned chronotherapy for BP, few cardiovascular studies have controlled for ToD, despite compelling evidence in preclinical models and the prominent call to action by chronobiology-aware cardiac physiologists [169,173,174]. Nonetheless, ToD is not a flawless proxy for individuals’ endogenous circadian rhythms. ToD should be considered in conjunction with other basic information about the organism (e.g., sex, status as a diurnal or nocturnal species, their sleep/wake cycles, engagement in night shift work or other forms of altered daily activity, variation in hormonal concentrations, etc.) and will inform the conclusions, and possible applications, of the published data.

## 9. Sex Differences in Cardiac Events

Pathological cardiovascular events can occur as a result of many factors that may be modifiable, such as diet, activity level, and alcohol and/or tobacco use, as well as other factors that are not modifiable, such as ethnicity and genetics. For example, sex is a non-modifiable biological variable that influences overall physiology and the body’s response to external stimuli. Only recently has sex been taken into account as a key variable informing incidence, prevalence, and outcomes. In this section we provide observations from human clinical and research data, as well as from nonhuman animal studies, that have informed our current understanding of the differences observed by sex.

### 9.1. Clinical Data and Human Studies

As mentioned, CVD is the leading cause of US deaths for both men and women. Although women suffer from CVDs, the data suggest that there are sex differences in the occurrence, severity, and outcome of these disorders. Most of the research into causes has been conducted on males [175,176]; similarly, development of interventions and treatments has also focused on males. Thus, understanding sex differences in the occurrence of cardiovascular events can improve prevention and treatment of these diseases [177,178,179,180]. Sex differences in typical and pathological cardiac function have been documented [181,182,183,184] in humans [184], and females generally fare better than males in response to cardiac insults. Although the mechanisms mediating these sex differences remain unspecified, evidence suggests that humoral signaling might be a factor.

The incidence of many cardiovascular disorders is typically higher for men compared to women; however the prognosis for women is poorer, as the onset of these diseases typically affects older, post-menopausal women. Coronary artery disease (CAD), also referred to as atherosclerosis, occurs when plaque accumulates around the arteries, significantly narrowing the arterial walls and impeding proper blood flow. In patients aged <55, males are at a higher risk to develop this condition [185]; however, females’ risk rapidly increases past the age of 60, reaching an equivalent risk to males over the age of 80. Nevertheless, risk factors such as hypertension, high BMI, inactivity, stress, smoking, and diabetes can alter one’s predisposition to develop CAD.

Sex differences seem to be dependent, at least partly, on hormonal states, as pre-menopausal (as opposed to menopausal) women have better outcomes than sex-matched controls following MI [186]. Pre-menopausal women present with a lower incidence of coronary artery disease and left-ventricular hypertrophy, as well as decreased cardiac remodeling. Pre-menopausal women also exhibit improved vascular function and lipid profile, both of which could explain the lower incidence of atherosclerosis and type II diabetes in females of child-bearing age [187]. These differences have been associated with estrogen and estrogen receptors (ER) [188]. However, hormone replacement therapy utilizing an estrogen-progesterone combination failed to improve outcomes in post-menopausal women, and unexpectedly increased their risk of experiencing a cardiovascular event [189]. Further, polymorphisms in ER differentially affect outcomes in men and women, albeit both increase their risk of MI [190,191]. Taken together, these data suggest that other factors might be at play. Indeed, it is likely that the differential effects of estrogen on cardio-protection are mediated by one or more signaling pathways, as estrogen can bind to both extra- and intra-cellular receptors in various cellular compartments, thus activating a multitude of signaling pathways [183,188]. Further, evidence suggests that ER subtype expression is influenced by sex [192]. Alternative hypotheses to address sex differences in cardiovascular events, such as differences in Ca^2+^ permeability and mitochondrial dysfunction, have also been proposed and reviewed elsewhere [182], and are beyond the scope of this review.

Research examining sex differences in outcomes following cardiac arrest (CA) reports mixed results between males and females, even when protective measures such as therapeutic hypothermia (TH) are used. TH has long been used as a neuroprotective measure at the onset and duration of ischemic events [193,194,195]. A retrospective study examining sex differences in recovery from CA determined that men were more likely to have better general cognitive outcomes than their counterparts, whereas women were more likely to exhibit depressive mood, although these sex differences were not observed if patients underwent therapeutic hypothermia [196], which presumably reduced the CA-induced cellular damage. Another study analyzing outcome differences following CA with TH reported that there is a sex-modified effect of TH on neurological outcomes, with men having better neurological outcomes [197]. The association between TH and recovery differed by age, sex, and cardiac rhythm (shockable vs. no-shockable rhythm), and male patients under the age of 45 demonstrated elevated neuroprotective effects.

Despite the increasing body of knowledge addressing sex differences in cardiovascular events, some challenges with regards to clinical research remain. First, it is complicated to differentiate between sex differences due to variations in the cause of or type of cardiovascular event from sex differences in cardiac sensitivity within the myocardium and cardiomyocytes themselves. Second, not being able to identify environmental and other factors that might contribute to the individual and specific events further confound the interpretation of results. Moreover, the seemingly contradictory effects of estrogen replacement therapy on post-menopausal women demonstrate the complexity and potentially, yet-to-be identified interplay between hormones and secondary mechanisms. Further, sex differences in the prevalence of risk factors of CVDs, such as stress and inflammation, further contribute to the complexity in the etiology and development of these diseases. Finally, these studies failed to report the ToD of the events. As established before, daily rhythms exist in cardiovascular and all physiological processes, and, consequently, fluctuations in hormones, as well as endothelial, pro-thrombotic, and anticoagulant factors, can greatly influence cardiac event onset and subsequent outcomes. In sum, many factors contribute to the development and persistence of cardiovascular diseases, and although all factors do not affect all individuals in the same manner, establishing and standardizing some reference markers will promote the progress towards holistic and, eventually, personalized medicine.

### 9.2. Animal models

Animal models of disease states have proven valuable tools to isolate and study specific physiological processes in a more highly controlled environment than is possible in clinical studies. Different species and animal models contribute unique insights into physiological function and dysfunction, and have demonstrated translatability to human physiology. Animal models of CAD include pigs, rabbits, non-human primates, and rodents (reviewed in [198]). In a non-human primate model of atherosclerosis, animals fed a high cholesterol diet developed significant coronary artery atherosclerosis compared to controls, and males had significantly higher atherosclerotic deposits than females [199]; however, females that were ovariectomized had an accelerated development of atherosclerosis [200], suggesting that ovarian hormones might play a role in the effects observed. Another group reported that female rabbits fed a high cholesterol diet developed mild plaque deposits in the lumen of the aorta (~10%), whereas in males, plaques covered 42% of the lumen [201]. They concluded that higher nitric oxide release from the female aortic endothelium likely contributed to protection from an exacerbated atherosclerosis. These studies lend evidence to the hypothesis that a predominantly female hormone may play a role in cardio-protection, and loss of this hormone may accelerate the progression of cardiovascular pathology.

Data from MI models have provided mixed results. Following an ischemic event, increased resistance of the myocardium to I/R injury is observed in female mice, rats [202,203], rabbits, and dogs [183]; however, in female white New Zealand rabbits, this was not the case [204]. When the cardio-protective drug diazoxide (a mitochondrial ATP-sensitive channel opener) along with K/Mg cardioplegia were used, both sexes exhibited better outcomes than their control counterparts. However, age (>32 months, ‘aged’), but not sex, had an effect on recovery and infarct size, with male white New Zealand rabbits having better outcomes, even with both cardioprotective measures, than females [203]. This study also determined that there was an interaction of age by sex on post-ischemic recovery and infarct size, suggesting that age is a stronger predictor of outcome than sex in this species. However, aged female white New Zealand rabbits are in diestrus until induced to ovulate, which would indicate that estradiol might play a role in these findings, which is in line with prior studies [205]. A similar I/R study in Sprague Dawley rats reported that sarcolemmal ATP-dependent K^+^ channels are necessary for cardio-protection in females, significantly reducing infarct size [202] and blocking them during the ischemia, but not in the reperfusion phase, which significantly increased infarct size. A limitation of this study was that they did not test both adult and aged rats (nor did they specify the age of the rats used), and, hence, exploring sex differences in relation to hormonal changes was not possible. Finally, it should be noted that both of these studies were conducted ex vivo, and hearts underwent an I/R protocol using a Langerdorff apparatus. Thus, the conflicting findings among various mammalian models complicate the conclusions that can be derived from this work and, consequently, the potential translatability to clinical settings.

Studies focusing on the effects of estrogen seem more aligned with observations in the human population. Mirroring human data, estrogen is protective for young female rats, but its effects disappear with age or ovariectomy [204]. Estrogen also accelerates heart regeneration by promoting the inflammatory response in female zebrafish [206], and these effects extend to males when treated with estrogen. Upon reperfusion, female rat cardiomyocytes returned and exceeded baseline cardiac contraction amplitude while males’ remained significantly reduced, and only 40% of male myocytes were viable, compared to 100% in females [204]. Furthermore, when female, ovariectomized rabbits consuming a high cholesterol diet are provided with estrogen supplementation, these rabbits are protected from atherosclerotic development compared to ovariectomized females not treated with estrogen [207]. Despite these and other data lending support to the hypothesis that estrogen is a protective agent, the particulars of how estrogen affects both females and males, and how the data could be translated to the human population, remain to be seen.

Animal models of cardiac disease allow us to isolate specific aspects of cardiac malignancies, having both advantages and limitations with regards to the manipulation of various pathogenic aspects—lipid profile, elevated BP, risk factors, and their course to pathology—with each contributing some understanding of the hows and the whys underlying pathology. They have greatly contributed to our understanding of the differences in the occurrence of these events, and how they differentially affect organisms, as well as if and how sex differences contribute to outcomes. Nevertheless, as with human data, animal models report varying findings when it comes to sex differences in pathological cardiac events and outcomes, which complicates the interpretation of the data. Further confounding interpretation, and the potential translatability of the published data, are the differing findings across species, which cast some doubt on how the results may apply to therapies for humans. Here, we have compiled merely a few of all the animal studies available that address sex differences within the context of cardiovascular disease, with the goal of highlighting some of the most salient and consistent findings in the field. However, it is not meant to be an exhaustive review, nor to imply that sex as a biological variable has been adequately and rigorously taken into account in the cardiovascular health literature. For a more comprehensive understanding of this topic, please refer to [184,198,208,209,210].

## 10. Conclusions

Circadian rhythms are present in all mammals and regulate many physiological processes. The cardiovascular system is not the exception. We have reviewed evidence detailing how this system is stringently regulated by circadian clocks, and how their disruption can have detrimental consequences on outcomes. Although relatively few studies have been conducted, we have presented human sample data that support and extend findings observed in rodent models. We have presented clinical data highlighting sex differences in cardiac event occurrence and outcomes, and pointed out the lack of consistent inclusion of females in preclinical models. Further, we have presented the importance of the ToD of cardiovascular events in terms of severity, response to therapeutic measures, and overall outcomes. Consequently, we highlight the relevance of including ToD as a biological variable, as this simple parameter can be easily reported, and then other relevant information regarding the organism’s circadian cycle can be inferred from it.

Technological and scientific advances that improve our understanding of human physiology and behavior support progress towards personalized medicine, in which clinical care becomes highly individualized. The consideration of temporal aspects of health will be an important component of this revolution in health care. Indeed, circadian variation in the delivery and/or absorption of medications has been reported (reviewed in [211]). This observation is supported by recent data demonstrating ToD-specific changes in the blood-brain-barrier permeability in *Drosophila melanogaster* [212], as well as xenobiotic efflux in the blood-brain barrier of mice and among cultured human brain endothelial cells [213]. Together, these results substantiate the importance of consistently reporting ToD in preclinical studies that may be used to inform clinical practice.

We acknowledge interindividual differences in circadian rhythms, and thus do not propose a ‘one size fits all’ approach. The current common reporting practices in the field generally do not provide sufficient information about the interplay among circadian rhythms, physiology, behavior, and optimal cardiovascular functioning to provide reliable, evidence-based clinical recommendations. Indeed, elevating ToD to a widely reported biological variable should inform interpretations of a study and provide an important step in the development of chronotherapeutics. Again, we do not propose to generalize the conclusions of these studies to the entire population; rather, we propose to use the ToD data to establish guidelines for typical temporal relationships among physiology and behavior that would require future adjustments for an individual’s sex, preexisting conditions, and overall lifestyle. Thus, we encourage using ToD information as simple, but presumably insightful, data that will empower chronotherapeutics.

The overarching goal of this review is to emphasize the importance of circadian regulation in the cardiovascular system and bring attention to the current gaps in our knowledge. Research in this field aims to understand the function and dysfunction within the cardiovascular system, to improve health and wellbeing. Thus, it is imperative that moving forward, researchers consider experimental questions, design experiments, and report findings including the critical biological variables of sex and time-of-day in order to produce reliable, reproducible, generalizable, and clinically translatable data.

## Figures and Tables

**Table 1:** Effects of clock gene mutations on cardiovascular function and regulation.

Gene	Effect	Reference
*Per2* mutation	Reduced NO production; decreased vasodilatory prostaglandins and increased vasoconstrictors	[81]
Global KO PPAR𝜸	Decreased variation in diel heart rate; abolished regulation of mean arterial pressure and HR	[82,83]
Germline deletion of *Bmal1*	Abolished diel fluctuations in HR and BP; disrupted metabolism; impaired contractile function; cardiac damage; early cardiac aging	[84,85,86]
*Cry1/2* double deletion	Salt sensitive hypertension; enhanced baroreflex sensitivity	[87]
*Per1* deletion	Non-dipping hypertension in male, but not female mice; very low BP; elevated endothelin-1	[88,89,90]

## Data Availability

Not applicable.
